# Unsupervised Learning Based on Multiple Descriptors for WSIs Diagnosis

**DOI:** 10.3390/diagnostics12061480

**Published:** 2022-06-16

**Authors:** Taimoor Shakeel Sheikh, Jee-Yeon Kim, Jaesool Shim, Migyung Cho

**Affiliations:** 1 Department of Computer & Media Engineering, Tongmyong University, Busan 48520, Korea; sheikh@tu.ac.kr; 2 Department of Pathology, Pusan National University Yangsan Hospital, School of Medicine, Pusan National University, Yangsan-si 50612, Korea; artinus2000@naver.com; 3 School of Mechanical Engineering, Yeungnam University, Gyeongsan 38541, Korea

**Keywords:** supervised learning, unsupervised learning, computer assisted diagnosis, autoencoders, whole-slide imaging, classification

## Abstract

An automatic pathological diagnosis is a challenging task because histopathological images with different cellular heterogeneity representations are sometimes limited. To overcome this, we investigated how the holistic and local appearance features with limited information can be fused to enhance the analysis performance. We propose an unsupervised deep learning model for whole-slide image diagnosis, which uses stacked autoencoders simultaneously feeding multiple-image descriptors such as the histogram of oriented gradients and local binary patterns along with the original image to fuse the heterogeneous features. The pre-trained latent vectors are extracted from each autoencoder, and these fused feature representations are utilized for classification. We observed that training with additional descriptors helps the model to overcome the limitations of multiple variants and the intricate cellular structure of histopathology data by various experiments. Our model outperforms existing state-of-the-art approaches by achieving the highest accuracies of 87.2 for ICIAR2018, 94.6 for Dartmouth, and other significant metrics for public benchmark datasets. Our model does not rely on a specific set of pre-trained features based on classifiers to achieve high performance. Unsupervised spaces are learned from the number of independent multiple descriptors and can be used with different variants of classifiers to classify cancer diseases from whole-slide images. Furthermore, we found that the proposed model classifies the types of breast and lung cancer similar to the viewpoint of pathologists by visualization. We also designed our whole-slide image processing toolbox to extract and process the patches from whole-slide images.

## 1. Introduction

Currently, pathologists use whole-slide images (WSIs) to make an accurate primary pathological diagnosis. A manual histopathological examination of such images is time-consuming, and it is difficult to reproduce them; thus, an efficient and robust automated system is needed that can achieve quick scanning and diagnosis of potential disease regions. With the advancement of digital imaging techniques and the advent of WSIs, significant attention has been devoted to the automatic detection and classification of cancers. Current research in this field focuses on machine-learning- and deep-neural-network-based methods, directly impacting clinical and related studies, as well as the progression and development of targeted therapy approaches [1]. Computational tools based on the digital slide workflow concept can help increase the efficiency and accuracy of detection while enabling a more detailed and precise disease analysis.

The challenges in deep learning for cancer or disease diagnosis on histopathological images include the use of large amounts of accurately annotated data and the generalization limitations of WSIs due to multiple variants and the intricate cellular structure of histopathology data. Publicly available WSIs with accurate annotation or label information are fewer as compared to natural image datasets because acquiring WSIs is a difficult and costly task owing to the specific nature of medical imaging modalities [2,3,4,5]. To address this issue, previous researchers used pre-trained models based on previously acquired knowledge from different domain image datasets and then applied them to the medical imaging domain.

However, for rare types of cancers, it is difficult to obtain WSI data. We thought it would be a very effective idea to use multiple descriptors such as HOG and LBPs for shape and texture feature extraction, respectively, which not only showed improved performance together [6], but can help obtain sufficientinformation when the number of data is very small. We propose an unsupervised learning model for heterogeneous feature learning for WSI classification, which showed relatively good performance even on small balanced datasets and has not yet been exploited. Our simple structured model is superior in the sense that we do not assume a specific set of pretrained features based on different classifiers to achieve high performance. Instead, we rely only on unsupervised latent spaces learned from stacked autoencoders based on any number of independent multiple descriptors, which enables the model to extract and fuse these rich spaces and can be used with different types of classifiers. Our model can classify the rare types of cancer diseases from WSIs more accurately than previous literature methods.

The contributions of our paper are as follows:

(1) We propose an unsupervised deep learning model that uses stacked autoencoders to learn the multiple low-dimensional latent spaces from an input, including the histogram of oriented gradients (HOG) [7] and local binary patterns (LBPs) [8]), along with the original image to fuse the heterogeneous features. Two additional inputs as descriptors help the model learn and classify multiple variants’ cellular information robustly from less or partially available annotated data. Although only three image descriptors, which are raw images and their HOG and LBP, representations are assumed as the inputs, we can use as many as required for future research.
(2) Our model outperforms the existing state-of-the-art models, yielding the best score for binary-class and multi-class classification using four metrics, and it achieved the highest overall multi-class accuracies and other metrics for two public benchmark WSI cancer datasets.
(3) We also discuss the performance of the model from various perspectives using multiple-input descriptors, visualization experiments such as confusion matrices, and the t-SNE technique, which confirmed that our model classifies the WSIs similarly to the way pathologists diagnose. The AUC (ROC) curves of the model also showed our model can be used clinically for cancer diagnosis.

The remainder of this paper is organized as follows. In Section 2, we discuss related research. The architecture and technical details of the proposed model are presented in Section 3. Section 4 describes the datasets used in this study. The experimental setup, model training, and implementation details are described in Section 5. Section 6 and Section 7 provide a detailed comparison of the classification results of WSIs and examine these results from the perspective of pathological diagnosis by visualization the last layer of the model. Finally, Section 8 provides concluding remarks and potential areas for future research.

## 2. Related Work

With the continuous technological advances in digital scanners and image visualization methods, the diagnosis of disease through glass slides manually is rapidly replaced by WSI inspection based on AI algorithms. These changes are expected to further accelerate the development of automatic diagnosis systems through WSIs; as the development of AI techniques progresses, such methods assist pathologists in making early decisions on treatments.

Hand-crafted features and clustering techniques have been extensively researched, such as [9], which extracts the handcrafted features from annotated tumor regions of WSIs and then applies a machine learning model for the top feature selection and cancer classification, which solely relies on careful annotations of cancer regions of WSIs. Balazsi attempted to overcome the effect of fixed tiling of WSIs by using color, texture, and gradient features extracted with RF classifiers for invasive ductal carcinoma versus normal classification [10]. Barker applied the unsupervised K-means technique on phenotypes of patches, followed by feature selection via CNN and feature aggregation for the final prediction of features that represent the morphological character, texture, and statistical property of malignant tumors [11]. Spanhol designed a method to extract the image patches from the BreaKHis dataset for training the CNN and, later, the combination of these patches for final classification. The performance metrics showed improved results when compared to previous models trained with handcrafted textural descriptors [12]. In contrast, Zhu obtained the survival prediction by utilizing only the WSI-level labels [13]. Whitney developed an algorithm to provide quantitative measurements of nuclear shape and size, which could be applied across different tumor subtypes [14]. Bahlmann used color gradients of square patches with linear SVMs for the classification of diagnostically relevant versus irrelevant regions in WSIs, but used only a small set of patches [15]. Bejnordi used a 2D long short-term memory to aggregate the context information of WSIs from a grid of neighboring patches and classify spatial information [16]. Litjens designed a deep network with 128 × 128 pixel patches at 5× magnification for the delineation of prostate cancer [17]. Bejnordi proposed a context-aware stacked CNN to take advantage of spatial information within WSIs [18]. Lin built a framework by leveraging a fully convolutional network for efficient inference while reconstructing dense predictions to ensure detection accuracy [19]. Cruz-Roa proposed a three-layer CNN that operated on 100 × 100 pixel patches at 2.5× magnification, which was more successful than color-, texture-, and graph-based features with an RF classifier in the detection of invasive breast cancer regions [20]. Attallah proposed a model for the diagnosis of pediatric medulloblastomas and their subtypes from WSIs. The textural analysis features and deep learning extraction methods were used in a cascaded manner, and the best accuracy results were reported for binary and multi-class classification [21]. Attallah and Zaghlool applied a combination of textural and deep learning features to improve subtype identification of WSIs. This technique yields an increase in the accuracy of the classification of pediatric medulloblastomas with a relatively small number of features [22]. Anwar designed a CAD method, which includes a fusion of features extracted from the ResNet CNN model combined with features of wavelet packet decomposition (WPD) and HOG. Later, these features were reduced by the PCA technique and achieved the highest accuracy of 97.1% [23]. Attallah proposed a Histo-CADx model, which applied the fusion of different deep learning techniques with handcrafted feature methods to learn the set of fused features. Then, a classifier system with the fusion of outputs from different classifiers was used, which showed improved accuracy results with BreakHis and the ICIAR 2018 datasets [24].

A few weakly supervised methods have also been presented. For example, Dundar used multiple instances of learning for discrimination of benign cases from actionable (atypical ductal hyperplasia and ductal carcinoma in situ) by using WSIs with manually identified ROIs [25]. Sudharshan proposed a framework for the diagnosis of breast cancer patients, to investigate the relevance of multiple instances’ learning with sample images into bags (patients) without the need to label all the instances. The experiments were conducted on the BreaKHis dataset and achieved the highest results with 40× magnification for both images or patientswise cases [26]. Some additional studies have used multiple instance learning [27,28] to address the classification problem by automatically extracting valuable information from coarsely labeled patches. Some studies also used multiple resolutions [29], multi-field-of-view sliding windows [30], a range of magnification levels of WSIs [31], and high-level WSI classification tasks [32,33] to analyze WSIs and reported accurate survival predictions [34].

Literature studies have used supervised or weakly supervised learning methods or proposed fused models for a more accurate diagnosis of WSIs. The fused models [35,36] have demonstrated better computational performance than the specific set of optimal feature extractors, but these models are more dependent on the selected set of features or classifiers, which might not work with relatively rare types of cancer cases, extracted from WSI data. In contrast, our study helps to obtain sufficient information from WSIs, by using any number of independent multiple descriptors for unsupervised learning of features, and later, different classifiers (supervised) can be used to achieve high performance for any type of WSI classification. The fusion of latent spaces learned with multiple descriptors extracts more distinct and salient information while retaining coarse-scale features of WSIs at specific magnification levels. Our model also consists of separable modules that can be used for various medical image analysis tasks.

## 3. Proposed Model and Architecture

**Figure 1:**
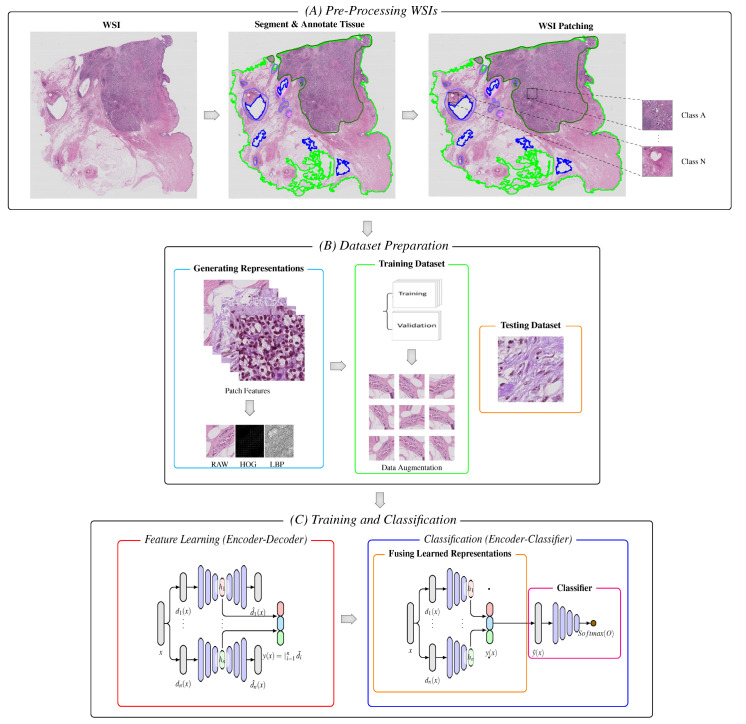
Block diagram of the proposed model. (**A**) Following segmentation and annotation, image patches are extracted from the tissue regions of the WSIs. (**B**) Patches are encoded into a set of descriptive feature representations (i.e., RAW, HOG, and LBPs). The representations are further split into two portions, and data augmentation is applied to the set of descriptive representations of the training portion. (**C**,**Left**) For unsupervised learning, the set of generated descriptive features is feed into the stacked autoencoders without labels, which embeds the input vectors into a lower-dimensional space. (**C**,**Right**) The learned representations are fused together and passed with labels to the classifier with respective labels, which are used to make the final diagnostic prediction and classification.

The block diagram of the proposed model is schematically shown in Figure 1. The model consists of a WSI pre-processing stage, data representation stage, and two main processing sub-blocks of the training and classification stages. The latter includes the unsupervised learning of multiple features using stacked autoencoders [37], the fusion of hidden latent spaces, and the final classification layers.

### 3.1. Pre-Processing WSIs

#### 3.1.1. Segment and Annotate Tissue

For each slide, our pipeline begins with automated segmentation of the tissue regions, and we developed our own pre-processing WSI system to obtain patches from WSIs. The WSIs are read into memory at a down-sampled resolution, for example, 64×, converted from RGB to the HSV color space. A binary mask for the tissue regions is computed based on the threshold of the saturation channel of the image after median blurring to smooth the edges; subsequently, we applied an additional morphological closing operator to fill small gaps and holes. The approximate contours of the detected foreground objects are then filtered based on an area threshold and stored for downstream processing, while the segmentation mask for each slide is made available for other visual analysis in the next work. A human-readable file is also generated, which includes the list of files processed with important fields containing the set of segmentation parameters used. The segmentation parameter list includes a segment level that represents the down-sampled level of WSIs, which can be easily loaded into memory for segmenting and annotating classwise regions, thresholds, and so on.

#### 3.1.2. WSI Patching

After segmentation, our algorithm crops 224 × 224 patches from within the segmented foreground contours at the user-specified magnification and stores stacks of image patches along with their coordinates, class labels, and slide metadata using the hierarchical data format (hdf5). Depending on the size of the WSIs and the user-specified magnification, the number of patches extracted from each slide range can vary from hundreds to hundreds of thousands. The number of extracted patches for each class was also saved in separate folders with the respective classwise label information.

### 3.2. Dataset Preparation

#### 3.2.1. Generating Representations

The next important phase after WSI patching is extracting a set of n feature descriptors from the set of extracted patches. The main reason for using these feature descriptors is to learn the overall variant structure of the cells, such as the color, shape, and texture. We used two feature descriptors, HOG and LBPs, to extract the shape and texture features, respectively. This controls the significant loss of visual information that disappears during learning, which results in poor classification performance [38]. We re-scaled the patch raw images to a downscaled ratio (40 × 40) to enhance the finer visually important details and kept the size of the images in each category intact. In the next step, we further divided the input data (x) into two sub-modules: training and testing.

#### 3.2.2. Training Dataset

The training data without augmentation were subdivided into training and validation sets. We generated an augmented dataset from a subset of three representations: RAW, HOG, and LBP. In general, pathologists study histological images from various orientations and magnifications, and there are many variations in the staining and acquisition conditions. We applied different data augmentation techniques to mimic the pathologist examination process and realistic variations, such as flipping (horizontal and vertical), rotation, shifting (width and height), brightness, zooming, and blurring. Such data augmentation can increase the size of the dataset without deteriorating its quality. These images were used to train the model from scratch without any pre-trained weights.

#### 3.2.3. Testing Dataset

The remaining portion of the data was used to validate the performance of the proposed model.

The data were flattened into vectors from images before feeding the data to the training and classification modules because we used multi-layer autoencoders that accept a vector as the input.

### 3.3. Training and Classification

This is the main stage of our model, which includes a hierarchical feature contraction and fusion scheme based on autoencoders for classification.

#### 3.3.1. Feature Learning

To learn multiple representations from input patches x, we used a set of different image descriptors $M_{f}$ that included three different characteristics (color, shape, and texture), represented as $M_{f}={\left\{d_{i}\left(x\right)\right\}}_{i=1}^{n}$, where $d_{i}\left(x\right)$ represents the feature vector of each representation. We wanted to convert data x into a more suitable representation that has particular distributions or characteristics, making it easier to handle. Several methods can be used to achieve a compact space, including clustering or dimensionality reduction, followed by a mapping function. Nevertheless, autoencoders demonstrated all of these capabilities using a single method [39]. They learn an identity function by embedding input vectors into a lower-dimensional space that learns the relevant features. Owing to this characteristic, they are the backbone of our proposed architecture.

Our model consists of a set of n autoencoders, $H={\left\{h_{i}^{\theta_{i}}\right\}}_{i=1}^{n}$, parameterized by $\theta_{i}\in \Theta$, where Θ is the set of parameters for the complete model. Hence, for the raw image patch along with its corresponding HOG and LBPs, there should be 3*n* features learned for feature fusion. In the following text, we suppress the parameters from the notation of each AE for a simplified and concise representation. The goal of each AE is to compress input $d_{i}$ into a more expressive space, that is ${\hat{d}}_{i}\left(x\right)=h_{i}\left(d_{i}\left(x\right)\right)$, where the ith reconstructed feature ${\hat{d}}_{i}\left(x\right)$ is the output of the ith AE $h_{i}$ that operates on the input feature $d_{i}\left(x\right)$. During the training of each AE, the objective was to minimize the error between the reconstructed and original features. Hence, we define our feature reconstruction loss as

(1) $$L_{func}=\sum_{i=1}^{n}||{\hat{d}}_{i}\left(x\right)-d_{i}\left(x\right){\left|\right|}^{2}$$

#### 3.3.2. Classification

We need a compact representation learned within the autoencoders, because they define a space that separates and clusters the input learned data [40]. In general, the ith AE, $h_{i}$, is the composition of encoder $E_{i}$, and decoder $D_{i}$ functions such that $h_{i}\left(d_{i}\left(x\right)\right)=D_{i}\left(E_{i}\left(d_{i}\left(x\right)\right)\right)$. Let the hidden compact representation of the ith AE (the output of the encoder) be represented as ${\tilde{d}}_{i}\left(x\right)=E_{i}\left(d_{i}\left(x\right)\right)$. We are then interested in the learned feature representation $y\left(x\right)={\mathrm{error}}_{i=1}^{n}{\tilde{d}}_{i}\left(x\right)$, where error is the concatenation operator. To find the optimal set of parameters Θ for our model, the loss of n autoencoders is minimized by the function defined in Equation (2), where the loss function in Equation (1) is parameterized by Θ, which corresponds to the parameters of each AE, $\left\{H^{\Theta }\right\}$, which were omitted in the previous subsection. These training parameters are learned through back-propagation of the training phase.

(2) $$\Theta^{*}=\underset{\Theta }{arg min}\left\{L_{func}^{\Theta }\right\}$$

At the final stage of the proposed model, we replaced the decoder stage with a classifier stage. Our classifier stage consists of fully connected layers, which are connected to the concatenated feature space representations y(x) of the encoders. Given any learned latent representations, y(x), we can extract the multiple feature representations to retrain our classification model with fused latent representations connected to the fully connected layers and with the help of labels of the training set to classify the samples after passing through the encoder and classifier layers. We present more details in Section 5 with regard to our experimental setup. Our model has a loosely coupled architecture that allows us to switch between various classifiers without the need to relearn the unsupervised feature representation learning process.

### 3.4. Model Architecture

With the following design considerations, we summarize the details of the architecture in Table 1:

(1) **Normalize (N):**This layer accepts the raw image to normalize the color distribution of an over-/under-stained image to a well-stained target image by using a simple H&E color normalization technique [41].
(2) **Input (I):** This layer accepts a normalized image, which is pre-processed further to extract features using two image descriptors, HOG and LBPs, corresponding to the image from the raw images H and L, respectively.
(3) **Feature Representation Set (FRS):** This operation is applied to flatten the output of each input image into feature vectors prior to feeding it to the network layers, which significantly reduces the layer operations and prepares the model input for learning.
(4) **Autoencoder Layers (AE-x):** Autoencoders are mainly dimensionality reduction algorithms with a couple of important properties: autoencoders are a specific type of feedforward neural network where the output is the mirror image of the input. Each AE consists of three components: an encoder, hidden layers (latent space), and a decoder. The encoder compresses the input into a lower-dimensional representation, which is known as a latent space, and then reconstructs the output from this representation. The tissues vary in the target images; thus, to explore different features, our model architecture has hidden layers with a sufficient number of operations to represent each of these features, as shown in Table 1.
(5) **Fused Feature (FF):** This operation, applied to concatenate the hidden learned representation of each AE hidden representation in the previous layer, significantly reduces the data handling and prepares the model for the final classification layers.
(6) **Output (O):** The number of output neurons corresponding to each class, which are normalized using the softmax function, depends on the type of classification. In the present study, we conducted a multi-class classification (eight or nine classes).

## 4. Datasets

We used the following two public WSI datasets as reference standards for training and evaluating our unsupervised deep learning model. These datasets contained different variants of cancer diseases collected from different patients.

### 4.1. ICIAR2018

**Figure 2:**
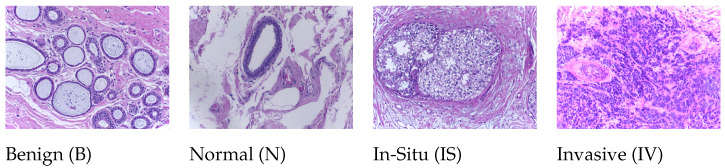
Microscopic H&E patched images of four types in the ICIAR2018 dataset.

This dataset (publicly available at https://iciar2018-challenge.grand-challenge.org/Dataset; accessed on 10 May 2019) is a version of the Bioimaging 2015 breast histology classification challenge dataset [42]. The dataset consists of 30 WSIs, where 10 are pixelwise labeled and 20 are non-labeled slides. These slides were digitized under the same acquisition conditions as Leica SCN400; the magnification was 20×, and the pixel dimensions were 0.467 μ per pixel. We used our own WSI pre-processing toolbox to generate patchwise images from each WSI based on the pixelwise annotations of WSIs and categorized them into one of four classes: (1) benign, (2) in situ, (3) invasive carcinoma, and (4) normal; for each case, the assigned class corresponds to a predominant cancer type in the respective WSI, as shown in Figure 2.

The goal of this challenge was to provide an automatic classification of WSIs into four classes. For our experiments, we used 10 labeled WSIs from which we generated a total of 32,215 patch samples, as shown in Table 2. Since the number of patches in the invasive class was more than approximately 10-times larger than the number of patches in other classes, there was a serious data imbalance. It is known that the unbalances in datasets can affect performance. Therefore, we created another balanced dataset that randomly selected only 3800 samples of the invasive class. We performed experiments on both datasets and looked at how much impact balancing the datasets had on performance. We ensured patient-based separation between the training and testing data to evaluate the performance of our model for clinical situations.

### 4.2. Dartmouth Lung Cancer

**Figure 3:**
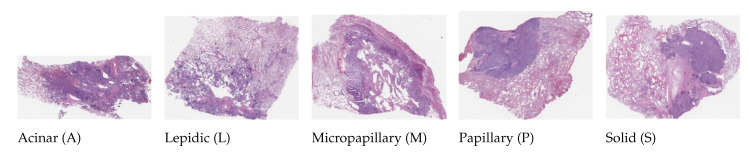
Histological images of five types of lung adenocarcinoma in the Dartmouth Lung Cancer dataset.

This dataset (publicly available at https://bmirds.github.io/LungCancer, accessed on 4 March 2019) is composed of 143 WSIs of lung adenocarcinoma from the Department of Pathology and Laboratory Medicine at Dartmouth-Hitchcock Medical Center (DHMC) [43]. All WSIs were labeled according to the consensus opinions of the three pathologists. These histopathology slides were scanned using a Leica Aperio whole-slide scanner at 20× magnification. A total of 31 WSIs were used to generate image patches for each WSI and categorized into five classes. Each class contains different random samples and types: (1) acinar, (2) lepidic, (3) micropapillary, (4) papillary, and (5) solid. Some examples of histological images are shown in Figure 3. We used 31 random WSIs owing to our limited hardware resources. The structural details of this dataset are listed in Table 3.

## 5. Experimental Setup

### 5.1. Aspects of Performance Evaluation

We evaluated the performance of the proposed model with respect to five different aspects: classification of binary-class and multi-class samples, comparison with literature encoders, effect of multi-input descriptors, confusion matrices with feature visualizations, and ROC curve analysis, as described in Section 6.1, Section 6.2 and Section 7.1–Section 7.3. To assess the performance of our model in clinical situations, we ensured that the same patient data were not used simultaneously during the training and testing phases. We trained the model using 5-fold cross-validation on a subset of training samples to determine the best hyperparameters for the model. For our experiments, we used a random 80:20 partitioning of the each dataset into training and testing subsets.

We used multiple solid-state drives and hard drive storages to store the raw files of digital WSIs. To perform segmentation and patching of WSIs, we used the Intel Xeon CPUs and feature extraction and learning using an NVIDIA Quadro RTX 5000 on local workstations with 128 GB RAM. The proposed model pipeline was implemented in Python and takes advantage of imaging processing libraries, such as OpenSlide, OpenCV, and NumPy. We used the Keras deep learning library to load data and train our models. Our source code is publicly available at https://github.com/AIMILab/Diagnostics, accessed on 12 June 2022).

### 5.2. Best Hyperparameters

Our model comprises two modules. For feature learning, four hyperparameters were applied for each autoencoder: the number of hidden layers, number of neurons per layer, type of activation function, and type of reconstruction loss. The following seven hyperparameters were tuned for the classifier: the number of layers, number of epochs, learning rate, batch size, optimizer, and batch normalization. The best selected parameters of our model with the cross-validation technique are listed in Table 4, according to the abbreviations in Table 1.

For feature extraction from descriptors, we used the following hyperparameters, which demonstrated the best results. HOG parameters: cells per block (1,1), orientations (16), and pixels per cell (14,14); LBPs: method (uniform), number of patterns (16), and radius of circle (2). These both descriptors help our model learn and classify the input samples more accurately with substantial information.

### 5.3. Performance Evaluation Metrics

The performance of the proposed pipeline was evaluated using several evaluation metrics including accuracy, sensitivity, precision, $F_{1}$-score, and the area under the curve of the receiving operating characteristic AUC (ROC). Equations (3)–(6) were used to compute these metrics, where the true positives (TPs) are the number of correctly predicts samples as the positive class. False positives (FPs) represent the number of incorrectly predicted samples as the positive class. True negatives (TNs) are the number of correctly predicted samples as the negative class. False negatives (FNs) represent the number of incorrectly predicted samples as the negative class.

(3) $$Accuracy=\frac{TP+TN}{TN+FP+FN+TP}$$

(4) $$Sensitivity=\frac{TP}{TP+FN}$$

(5) $$Precision=\frac{TN}{TN+FP}$$

(6) $$F_{1}Score=\frac{2\;\times \;TP}{2\;\times \;TP+FP+FN}$$

## 6. Performance Results

### 6.1. Comparison with Deep Learning Models

Table 5 and Table 6 show the classification results of binary-class and multi-class classifications with different well-known classification models for the two datasets, ours and the state-of-the-art models for histopathology diagnosis. The accuracy of the balanced dataset increased to 90.4% when only two classes were considered, and our model performed better than the other models by a large margin for the ICIAR2018 dataset, as shown in Table 5. Our method also demonstrated the highest results for all the other metrics when compared with the other models. For multiple classifications, the accuracy of our model outperformed the state-of-the-art models on both datasets, as shown in Table 6. The accuracy increased to 87.2% for the ICIAR2018 dataset and 94.6% for the Dartmouth dataset when four and five classes were considered.

We also noted that the variants introduced in these databases pose a significant challenge for the descriptors we use because HOG and LBPs are not invariant to the perspective and intensity transforms of these two datasets. Nevertheless, the sensitivity, specificity, and $F_{1}$-scores of our model outperformed those of the other CNN models. Clinically, because multi-class classification is able to determine cancer subtypes, it is more important for pathologists. For both datasets, the results of our model were higher, which indicates that our model can be developed for clinical use. It should be noted that there is a difference in performance between the unbalanced and balanced datasets. The balanced dataset improved the performance by more than 4% for all the metrics. These results demonstrate the importance of using a balanced dataset for deep learning models.

### 6.2. Comparison with Different Deep Encoders

We compared the performance behavior of deep feature encoders with the proposed encoder. We replaced the unsupervised stacked autoencoders of our model with different deep encoders that were trained and fine-tuned on both datasets using imagenet weights and later used these learned features in the classification stage. Table 7 shows the results for both WSI datasets. For the experiments, we used the following five deep encoders of well-known CNN models: ResNet-50 [44], Inception-V3 [45], DenseNet-121 [46], MobileNet [47], RuneCNN [48], BreastNet [49], LiverNet [50], and HCNN [51]. We report the accuracy of all the experiments on five-fold cross-validation. The maximum accuracies of 77.9% and 86.5% were achieved by the fine-tuned encoder of the HCNN model. Similarly, other encoders also obtained lower accuracy results compared to the HCNN encoder. Thus, the empirical results show that the idea of using fine-tuned deep encoders of complex pathological data is not efficient enough, and neither improve the performance results when used with the imagenet weights or deeply layer structured encoders. The use of image descriptors with stacked autoencoders obtained higher accuracy as compared to features learned from deep encoders for both datasets. It should be noted that the proposed stacked autoencoders with descriptors are more effective than the deep and complex CNN-based encoders.

## 7. Discussion

### 7.1. Effect of Multiple Descriptors

**Figure 4:**
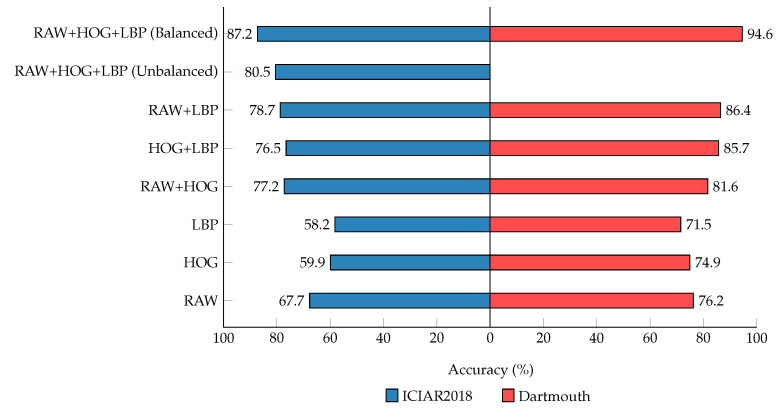
Accuracy for multi-class classification on the ICIAR2018 and Dartmouth datasets. Using the different combination of generated representations.

The behavior of our model for multi-class classification was thoroughly studied with special emphasis on factors that can affect performance. The number of inputs to autoencoders significantly affects the performance. In Figure 4, we observe that the performance improves as we feed more inputs to autoencoders in the form of image descriptors for both datasets. The input set of triplet representation shows the highest metrics in comparison to pairs or individual descriptors, which indicates that later fusion of learned representations for classification can increase the invariants of the given input descriptors. Our observation from the results is that removing the input combinations of descriptors will significantly degrade the results, as shown with the input set of single and pair representation. The performance can be further improved by employing more descriptors as inputs, which significantly boosts the metrics and classifies samples more efficiently. Based on our experiments, we concluded that our results align with the design of using multiple input descriptors as inputs to the stacked autoencoders and the later fusion of these learned latent spaces for classifying the sample of the WSIs more effectively. We believe that these results can also be applied to general medical images.

### 7.2. Pathologist’s Analysis of the Results

We analyzed the results of our model from a pathological point of view and confirmed that the results of our model are very similar to the diagnosis of pathologist’s. The performance of the ICIAR2018 dataset was slightly lower than that of the Dartmouth dataset due to the technical and the pathological point of view. The main technical reason arises from ambiguous data that are found in the set of patches for each class generated from the ICIAR2018 data by our WSI pre-processing system. We generated patches automatically using the label information provided. According to a pathologist, there are a number of patches that our system has made from areas designated as invasive, which should be classified as normal or benign. This is because we created a patch with a size of 224 × 224, which occupies more of the normal or benign than the invasive. However, in the Dartmouth dataset, such patches are extremely rare.

A pathologist said that the ICIAR2018 dataset has morphologic heterogeneity. This means the ICIAR2018 dataset comprises various spectrums of normal, benign, in situ, and invasive carcinoma. Even more, breast cancer is also morphologically heterogeneous in tumor types, such as invasive ductal, invasive lobular, mucinous, micropapillary, or invasive papillary carcinoma, and various histological grading scales, such as well-differentiated (more tubular formation) to poorly differentiated (more solid). In other words, each cluster of the ICIAR2018 dataset has a diverse spectrum of various diseases. The Dartmouth dataset includes only invasive carcinoma with a single histological subtype. Therefore, each cluster is a more homogenous composition compared to that of the ICIAR2018 dataset.

**Figure 5:**
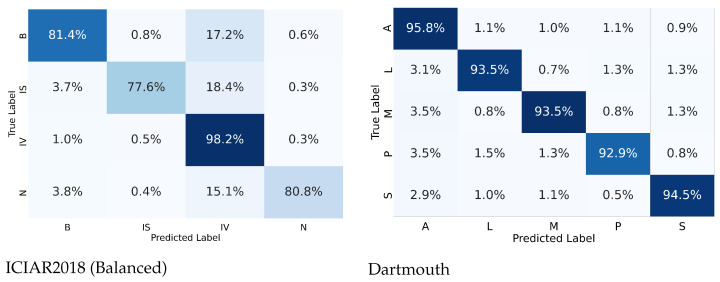
Confusion matrices for the multi-class classification of ICIAR2018 and Dartmouth datasets. Class labels are according to the abbreviations of Figure 2 and Figure 3.

From the confusion matrices shown in Figure 5, the ICIAR2018 dataset showed 18.4% differences of in situ carcinoma, which was interpreted as invasive carcinoma. This is because small patches of a training set of invasive carcinoma could contain scattered in situ lesions in between invasive areas, resulting in the deep learning being able to learn small patches of the in situ carcinoma as invasive carcinoma. In the real world, much invasive breast carcinoma, especially the luminal molecular subtype, has scattered in situ carcinoma adjacent to and mixed with an invasive component. However, carcinoma of the lung consists of purely invasive components with a very low proportion of in situ carcinoma. In lung cancer, in situ carcinoma itself is not a more common lesion than breast cancer.

**Figure 6:**
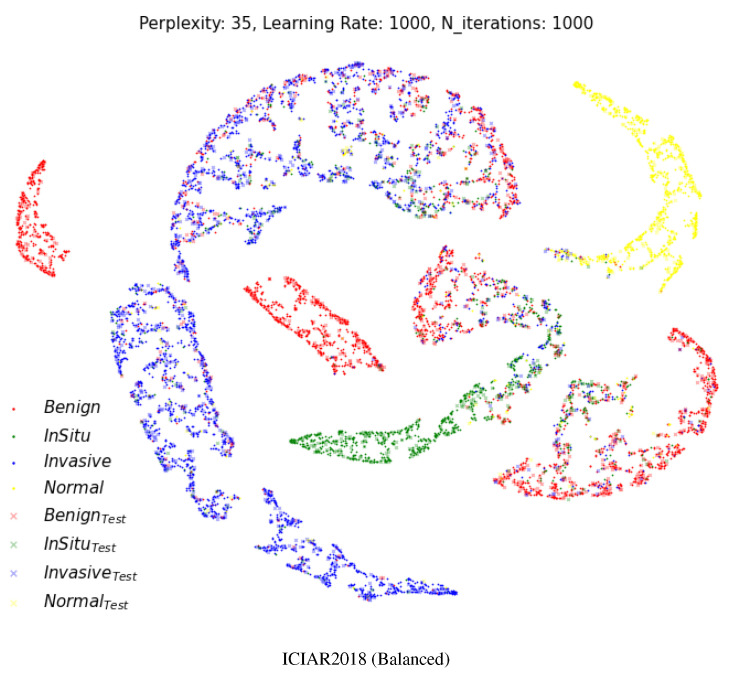

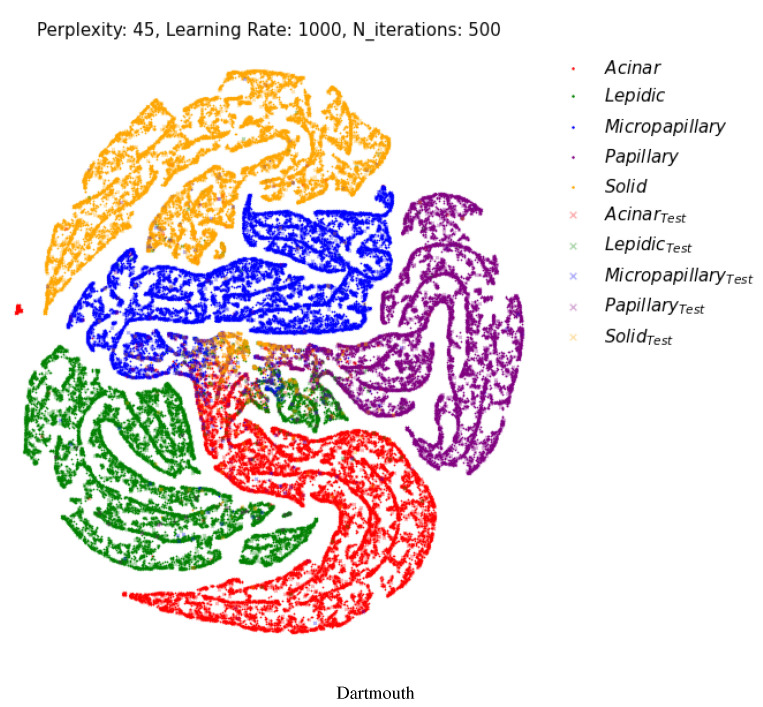
Two-dimensional visualization of two different layers using t-SNE [52] for the multi-class classification. Projection of the last fully connected layer. Cross shapes represent test samples. (Best viewed in color).

Figure 6 shows a two-dimensional visual representation of the activations of the last fully connected layer of the classifier stage. These representations result from the application of t-SNE [52], which is an efficient parametric embedding technique for dimensionality reduction that preserves the distance between samples. In these representations, each point corresponds to a sample instance, and the 2D distance between points is an approximation of the original Euclidean distance in a multidimensional space. In each row, the test datasets are represented by a cross symbol. The images appear in more organized and well-separated clusters for each class, indicating a good differentiation between images with different labels after the two fully connected layers of the model.

The visualization result shows that our model makes a very accurate and meaningful classification even from a pathological point of view. In Figure 6, the ICIAR2018 (Balanced) dataset has benign lesions (red) of the breast with four clusters. That is why benign breast lesions are not a single disease entity. Benign breast lesions encompass adenosis, ductal hyperplasia, cystic change, columnar cell change, columnar cell hyperplasia, papilloma, fibroadenoma, even atypical ductal hyperplasia, etc. Benign lesions composed of microglandular adenosis could resemble invasive carcinoma, or benign lesions composed of atypical ductal hyperplasia could have a morphologic similarity to in situ carcinoma. Compared to the various spectrum of benign lesions, normal, in situ carcinoma and invasive carcinoma are relatively homogenous disease entities.

In the Dartmouth dataset, two small isolated clusters (green and yellow) separated from the main clusters were identified, which were lepidic and solid, as shown in Figure 6. A small lepidic cluster was present in between acinar and papillary clusters. There could be two reasons for this. First, tumor type sub-classification is decided based on the main component of the invasive carcinoma, usually more than 90% of one subtype. In the case of more than 90% of acinar-type carcinoma and less than 10% of lepidic-type carcinoma, the main classification could be the acinar type. A smaller proportion of the lepidic type is really present in acinar-type carcinoma. The other reason is the morphologic similarities of each tumor subtype. Some patches of acinar-type carcinoma or papillary carcinoma share a resemblance with the microscopic features of lepidic-type carcinoma.

### 7.3. ROC Curves’ Visualizations

**Figure 7:**
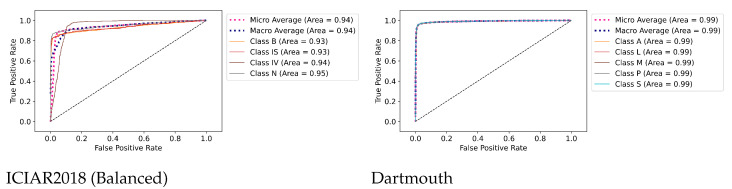
AUC (ROC) curves for the multi-class classification of the ICIAR2018 and Dartmouth datasets. Class labels are according to the abbreviations of Figure 2 and Figure 3 (Best viewed in color).

Figure 7 shows the AUC (ROC) results for the ICIAR and Dartmouth datasets. The AUCs of benign (B) and in situ (IS) of the ICIAR2018 dataset are 0.93, respectively, and the average is 0.94. The average AUC of the Dartmouth dataset is 0.99. This confirms that the proposed model can be used clinically for cancer diagnosis.

## 8. Conclusions

In this paper, we proposed an unsupervised deep learning model for WSI diagnosis using stacked autoencoders that learn and generalize different cellular heterogeneity representations from multiple-image descriptors, along with the original patches extracted from WSIs. Our model can use a set of N independent feature descriptors (i.e., currently, we used N = 3). We showed additional inputs help the model learn and classify multiple variants’ cellular information robustly on two publicly available WSI benchmark datasets. Our model outperformed existing state-of-the-art models by providing the best score for binary-class and multi-class classification using four metrics. The visualization results showed that the breast cancer and lung cancer classification are similar from the point of view of a pathologist.

The primary limitation of the model is deciding the size of patches that can learn more robust representations in the bottleneck space to separate the morphologically heterogeneous regions in sub-multi-level tumor types. We observed that this trend is due to the limited and unbalanced data, which affect the feature fusion stage. In future studies, we intend to make a more robust WSI processing toolbox to generate an accurate patch dataset for studying different feature descriptors to learn more about the WSI patch dataset. We will investigate the performance of the model on other feature descriptors that provide various variant comparisons and other datasets that provide diverse cancer cases. The proposed system can be adapted for diverse tasks associated with the domain of WSI-based diagnosis with relevance to clinical settings.

## Figures and Tables

**Table 1:** Proposed model architecture. H${}_{RAW}$, H${}_{HOG}$, H${}_{LBP}$∈ (Feature-X, Feature-X/K, K*Feature-X) with activation map. Abbreviations: Feature-RAW, Feature-HOG, Feature-LBP (Feature-X ∈), number of neurons (K), Number of hidden Layers (NL).

Block	Layers	Dimensions	# of Param.	Repetition
N	Input-N	N × N × 3	-	-
I	RAW	40 × 40 × 3	-	-
	HOG	8 × 8 × 1	-	-
	LBP	16 × 16 × 1	-	-
FRS	Feature-RAW	4800	23,044,800	-
	Feature-HOG	64	4160	-
	Feature-LBP	255	64,770	-
AE-R	Latent Vector${}_{RAW}$	H${}_{RAW}$	Varies	NL
AE-H	Latent Vector${}_{HOG}$	H${}_{HOG}$	Varies	NL
AE-L	Latent Vector${}_{LBP}$	H${}_{LBP}$	Varies	NL
FF	Concatenate Latent Vectors	5118	-	-
O	Dense	1024	5,241,856	-
	BN	1024	4096	-
	Dense	512	524,800	-
	Batch Normalization	512	2048	-
	Softmax	2/4/5	Varies	-

**Table 2:** Structure of the ICIAR2018 with 20× magnification factor.

Classes	Subtypes	# of Patches	
		Unbalanced	Balanced
Non-Carcinoma	Benign (B)	3770	3770
	Normal (N)	1200	1200
Carcinoma	In-Situ (IS)	1655	1655
	Invasive (IV)	25,590	3800
Total		32,215	10,425

**Table 3:** Structure of the Dartmouth Lung Cancer dataset (31 WSIs only) with 20× magnification factor.

Classes	# of WSIs	# of Patches
Acinar (A)	9	38,611
Lepidic (L)	5	39,092
Micropapillary (M)	5	40,349
Papillary (P)	4	32,228
Solid (S)	8	38,190
Total	31	188,470

**Table 4:** The best hyperparameters of our model.

Dataset	Parameters	Proposed Model		
		Autoencoder		Classifier
		Binary	Multi	
ICIAR2018	Number of Hidden Layers (NL)	1	1	-
	Number of Neurons (K)	Double	Same	-
	Activation Map	ReLU		-
	Epochs	200		30
	Learning Rate	e−4	e−3	e−4
	Optimizer	Adam		Adam
	Batch Size	64	128	32
Dartmouth	Number of Hidden Layers (NL)	1		-
	Number of Neurons (K)	Same		-
	Activation Map	ReLU		-
	Epochs	200		40
	Learning Rate	e−5		e−5
	Optimizer	Adam		Adam
	Batch Size	512		64

**Table 5:** Comparison of four metrics on the ICIAR2018 for the binary-classification. The best results are shown in **bold**.

Model	Metrics							
	**Unbalanced**				**Balanced**			
	**Accuracy**	**Sensitivity**	**Precision**	**$F_{1}$-Score**	**Accuracy**	**Sensitivity**	**Precision**	**$F_{1}$-Score**
Ours	**0.864**	**0.863**	**0.866**	**0.826**	**0.904**	**0.904**	**0.919**	**0.903**
ResNet-50	0.798	0.808	0.784	0.796	0.887	0.864	0.873	0.852
Inception-V3	0.833	0.843	0.807	0.824	0.864	0.874	0.853	0.840
MobileNet	0.763	0.668	0.771	0.716	0.831	0.842	0.825	0.827
DensetNet-121	0.861	0.861	0.843	0.852	0.884	0.875	0.856	0.837
RuneCNN	0.792	0.776	0.792	0.784	0.820	0.813	0.834	0.845
BreastNet	0.830	0.823	0.814	0.820	0.861	0.852	0.846	0.834
LiverNet	0.862	0.852	0.821	0.810	0.839	0.848	0.857	0.863
HCNN	0.854	0.859	0.835	0.842	0.885	0.863	0.854	0.871

**Table 6:** Comparison of four metrics on the ICIAR2018 and Dartmouth datasets for the multi-classification. The best results are shown in **bold**.

Dataset	Model	Metrics							
		**Unbalanced**				**Balanced**			
		**Accuracy**	**Sensitivity**	**Precision**	**$F_{1}$-Score**	**Accuracy**	**Sensitivity**	**Precision**	**$F_{1}$-Score**
ICIAR	Ours	**0.805**	**0.798**	**0.729**	**0.752**	**0.872**	**0.870**	**0.888**	**0.870**
	ResNet-50	0.789	0.705	0.681	0.693	0.851	0.843	0.864	0.850
	Inception-V3	0.697	0.744	0.724	0.734	0.840	0.861	0.842	0.834
	MobileNet	0.701	0.726	0.672	0.698	0.862	0.854	0.843	0.825
	DensetNet-121	0.707	0.658	0.672	0.665	0.854	0.861	0.873	0.852
	RuneCNN	0.642	0.608	0.656	0.631	0.832	0.824	0.827	0.834
	BreastNet	0.763	0.758	0.774	0.759	0.842	0.836	0.821	0.815
	LiverNet	0.784	0.776	0.754	0.763	0.856	0.861	0.843	0.827
	HCNN	0.791	0.784	0.773	0.788	0.853	0.855	0.851	0.842
Dartmouth	Ours	-	-	-	-	**0.946**	**0.941**	**0.942**	**0.941**
	ResNet-50	-	-	-	-	0.914	0.911	0.899	0.905
	Inception-V3	-	-	-	-	0.912	0.912	0.913	0.913
	MobileNet	-	-	-	-	0.884	0.909	0.873	0.891
	DensetNet-121	-	-	-	-	0.922	0.932	0.879	0.905
	RuneCNN	-	-	-	-	0.877	0.893	0.814	0.852
	BreastNet	-	-	-	-	0.901	0.934	0.926	0.921
	LiverNet	-	-	-	-	0.911	0.920	0.933	0.930
	HCNN	-	-	-	-	0.921	0.916	0.930	0.919

**Table 7:** Accuracy comparison of our encoder against literature encoders. The parenthesis values represent standard deviation.

	Dataset		
**Encoders**	**ICIAR**		**Dartmouth**
	**Unbalanced**	**Balanced**	
Ours	0.805 (0.022)	0.872 (0.022)	0.946 (0.019)
ResNet-50	0.742 (0.040)	0.854 (0.014)	0.710 (0.018)
Inception-V3	0.723 (0.035)	0.837 (0.009)	0.761 (0.052)
DensetNet-121	0.765 (0.022)	0.824 (0.037)	0.757 (0.019)
MobileNet	0.667 (0.060)	0.841 (0.043)	0.723 (0.013)
RuneCNN	0.641 (0.047)	0.838 (0.037)	0.672 (0.027)
BreastNet	0.788 (0.023)	0.856 (0.043)	0.826 (0.023)
LiverNet	0.767 (0.034)	0.859 (0.076)	0.871 (0.014)
HCNN	0.779 (0.067)	0.864 (0.034)	0.865 (0.017)

## Data Availability

Not Applicable.
